# Salvianolic acid C inhibits methane emissions in dairy cows by targeting MCR and reshaping the rumen microbial community

**DOI:** 10.1186/s40104-025-01285-8

**Published:** 2025-11-17

**Authors:** Zihao Liu, Li Xiao, Xiangfang Tang, Yue He, Xuemei Nan, Hui Wang, Yuming Guo, Benhai Xiong

**Affiliations:** 1 State Key Laboratory of Animal Nutrition and Feeding, Institute of Animal Science, Chinese Academy of Agricultural Sciences, No.2 Yuanmingyuan West Road, Haidian District, Beijing, 100193 People’s Republic of China; 2 State Key Laboratory of Animal Nutrition, College of Animal Science and Technology, China Agricultural University, No.2 Yuanmingyuan West Road, Haidian District, Beijing, 100193 People’s Republic of China

**Keywords:** Methane mitigation, Methyl-coenzyme M reductase, Rumen microbiota, Salvianolic acid C

## Abstract

**Background:**

Methane (CH_4_) emissions from ruminants significantly contribute to greenhouse gas effects and energy loss in livestock production. Methyl-coenzyme M reductase (MCR) is the key enzyme in methanogenesis, making it a promising target for CH_4_ mitigation. This study aimed to identify and validate plant-derived inhibitors by using molecular docking to screen compounds with strong binding affinity to the F430 active site of MCR and assessing their efficacy in reducing CH_4_ emissions.

**Results:**

Molecular docking analysis identified salvianolic acid C (SAC) as a potent inhibitor of MCR, showing a strong binding affinity to the F430 active site (binding energy: −8.2 kcal/mol). Enzymatic inhibition assays confirmed its inhibitory effect, with a half-maximal inhibitory concentration (IC_50_) of 692.3 µmol/L. In vitro rumen fermentation experiments demonstrated that SAC supplementation (1.5 mg/g DM) significantly reduced CH_4_ production (*P* < 0.01) without negatively affecting major fermentation parameters. Microbial community analysis using 16S rRNA sequencing and metagenomics revealed that SAC selectively altered the rumen microbiota, increasing the relative abundance of Bacteroidota while significantly reducing *Methanobrevibacter* (*P* = 0.04). Moreover, metagenomic analysis showed the downregulation of key methanogenesis-related genes (*mcrA *and *rnfC*), suggesting a dual mechanism involving direct enzymatic inhibition and microbial community modulation.

**Conclusions:**

These findings indicate that SAC effectively reduces CH_4_ production by inhibiting MCR activity and reshaping the rumen microbial community. As a plant-derived compound with strong inhibitory effects on methanogenesis, SAC presents a promising and sustainable alternative to synthetic CH_4_ inhibitors, offering potential applications for mitigating CH_4_ emissions in livestock production.

**Graphical Abstract:**

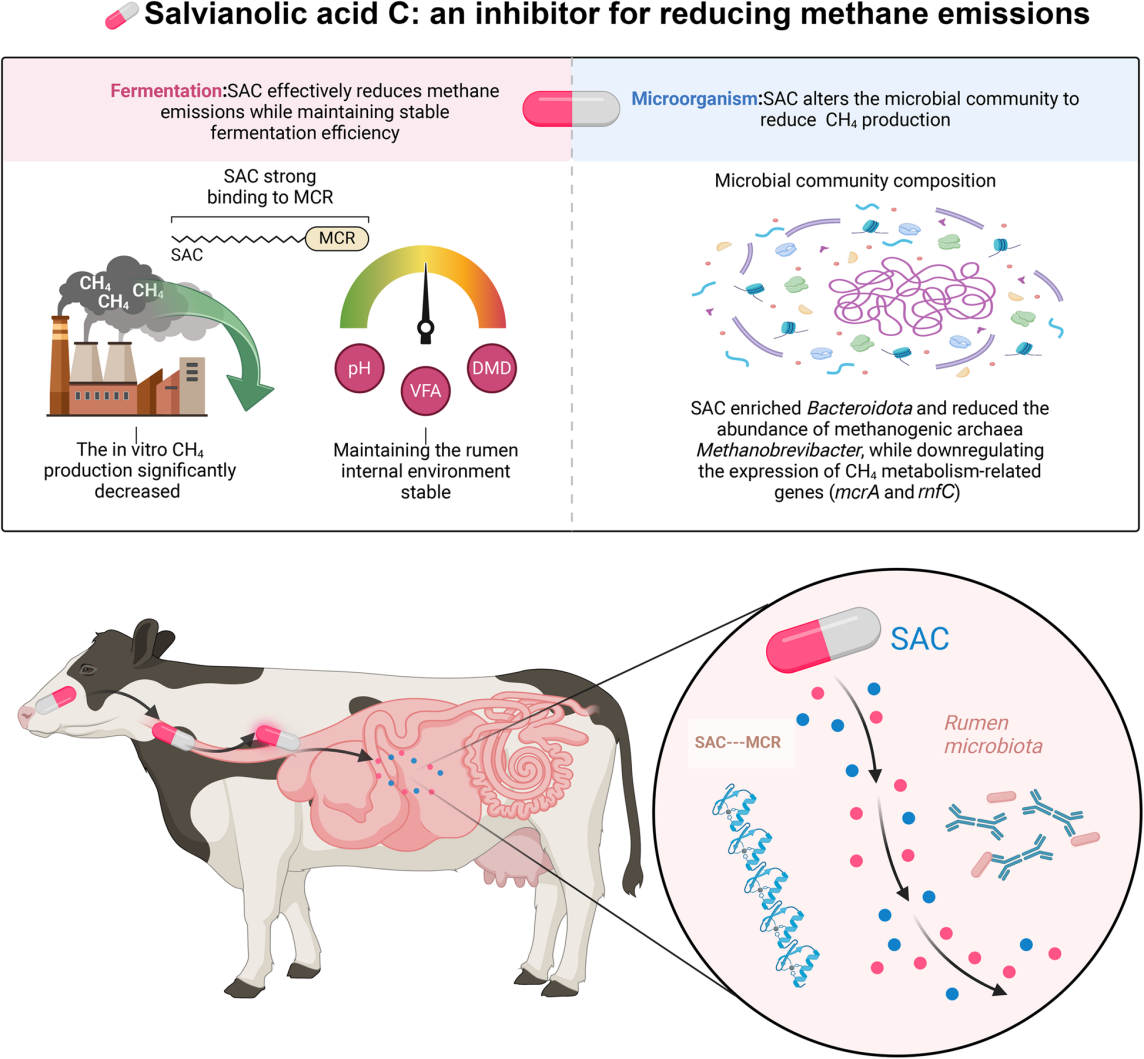

## Background

Since the beginning of the twenty-first century, global warming driven by greenhouse gas (GHG) emissions has become increasingly serious and has received widespread attention [1, 2]. Human induced GHG emissions come from diverse sources, including industry, transportation, agriculture, forestry and other land use, construction, and other energy related activities [3]. According to the Intergovernmental Panel on Climate Change (IPCC), the agricultural sector is considered the second largest source of anthropogenic GHG emissions globally [3]. It is estimated that in 2010, the agricultural sector accounted for approximately 11% of global anthropogenic GHG emissions [1, 4]. In the agricultural sector, the main GHG emissions are from livestock production [1, 5]. Methane (CH_4_) produced by ruminant enteric fermentation is a major source of GHG in livestock production, and its emissions continue to rise annually [3]. Ruminants possess a unique and complex rumen system, that enables them to convert indigestible plant fibers into volatile fatty acids (VFAs) and microbial proteins, which can be absorbed and utilized by the host [6, 7]. However, CH_4_ is a byproduct produced during the digestion process. It is the second largest GHG after carbon dioxide (CO_2_), with a global warming potential over 25 times greater than that of CO_2_ [8]. Furthermore, the emission of CH_4_ as a byproduct of the digestion process also results in energy loss [9]. According to statistics, about 6% of gross energy intake lost as CH_4_ [10]. Therefore, reducing CH_4_ emissions from ruminants is critical both for mitigating greenhouse effect and improving feed energy utilization efficiency.

The rumen is the primary site of CH_4_ production in ruminants, contributing up to 87% of total emissions [11, 12]. In the rumen, dietary carbohydrates are fermented and decomposed by microorganisms into VFAs, CO_2_ and H_2_ [13, 14]. Methanogenic archaea then utilize substrates such as acetate, formate, methanol, or CO_2_ and H_2_ to generate CH_4_ [15]. There are currently three main pathways known for CH_4_ production, namely the hydrogenotrophic pathway, the acetoclastic pathway, and the methylotrophic pathway [16]. Methyl-coenzyme M reductase (MCR) plays a pivotal role in all three pathways, as the final step of CH_4_ formation universally requires its catalysis [17, 18]. Specifically, MCR catalyzes the reduction of methyl-coenzyme M and coenzyme B to CH_4_ and heterodisulfide under anaerobic conditions [19]. This reaction process requires the presence of coenzyme F430 [20]. Coenzyme F430 is the active site of MCR, and its nickel center must be in the Ni(I) oxidation state for the enzyme to be active [21]. Therefore, inhibiting the activity of MCR is an effective strategy to reduce CH_4_ generation.

There have been studies reporting on some MCR inhibitors, such as 3-nitrooxypropanol (3-NOP), 2-bromoethane sulfonate (BES), and 3-bromopropane sulfonate (BPS). Both BES and BPS can inhibit the activity of MCR [22]. However, due to the susceptibility of BES to drug resistance and the inability of BPS to be transported to methanogenic cells [23], as well as toxicological reasons, their widespread application has been limited [24]. 3-NOP is an artificially synthesized compound that can inhibit CH_4_ emissions, but it also causes a significant increase in H_2_ emissions, and the subsequent metabolic fate of increased H_2_ remains unclear [25]. Plant extracts have a wide range of sources and rich functions [26]. Due to their safety profile, plant extracts have gained widespread attention as potential alternatives to chemical additives [27]. It has been found that some plant extracts could inhibit CH_4_ generation, such as saponins, tannins, and flavonoids [27]. However, challenges remain, including negative impacts on digestion and metabolism, potential toxicity, and inconsistent efficacy [27, 28]. Therefore, exploring specific plant extract inhibitors targeting MCR is crucial. At the same time, there are currently few studies that use high-throughput virtual screening methods and explore the mechanism of inhibitors in CH_4_ production.

This study aimed to target MCR and use molecular docking combined with high-throughput virtual screening technology to screen for a plant derived CH_4_ inhibitor with significant efficacy, and to elucidate its mechanism of action.

## Materials and methods

### Molecular docking and virtual screening

MCR is a multi-subunit enzyme complex, with the *mcrA*, *mcrB*, and *mcrG* genes encoding the α, β and γ subunit, respectively [29]. The 3D structure of MCR (PDB ID: 5G0R) was downloaded from the RCSB PDB database for molecular docking analysis. The Protein Preparation Wizard module was used to add hydrogens, remove the D/E/F chains, water molecules, and small molecules (TP7, AGM, DYA, GL3, MGN, MHS, SMC, etc.). The energy was then optimized (OPLS2005 field, RMSD 0.30 Å). The processed protein was converted into a grid file using the Receptor Grid Generation module, and the grid files were generated with small molecule F430 binding pocket as the center, and the box size was set to 20 Å × 20 Å × 20 Å. The 2D structures from the Natural Product Library (containing 3,900 compounds) were adopted by LigPrep Module of Schrödinger, which performs hydrogenation and energy optimization to output 3D structures for virtual screening. Virtual screening was conducted using the Virtual Screening Workflow module with the Glide docking program. Initially, compounds were docked to MCR using the high-throughput virtual screening (HTVS) mode, followed by standard precision (SP) docking. Subsequently, the top 15% of compounds based on docking scores were then subjected to extra precision (XP) docking. Finally, check the target and compound binding force, as well as the structure of the compound.

### Measurement of MCR inhibitory activity

MCR activity inhibited by plant extracts was measured using a microbial MCR ELISA kit (Meimian Industrial Co., Ltd., Jiangsu, China). All plant extracts were purchased from MedChemExpress (Shanghai, China) and dissolved in dimethyl sulfoxide (DMSO), then diluted to the desired concentrations using 50 mmol/L HEPES buffer (pH 7.5). In each well, 40 µL of MCR and 10 µL of plant extract (final concentration 100 µmol/L) were added. The plate was gently mixed and incubated at 37 °C for 30 min. After incubation, the liquid was discarded, excess liquid was shaken off, and the plate was washed. Then, 50 µL of enzyme substrate was added to each well, and incubation continued for another 30 min at 37 °C. Afterward, 50 µL of chromogenic agent A and 50 µL of chromogenic agent B were added to each well, followed by incubation for 10 min at 37 °C, shielded from light. The reaction was terminated by adding 50 µL of terminating solution, and the absorbance (OD) at 450 nm was measured using a microplate reader (Thermo Labserv K3 TOUCH, Thermo Scientific, Waltham, MA, USA).

Salvianolic acid C (SAC), a potent MCR inhibitor, was selected for further investigation based on its ability to effectively inhibit MCR activity. To determine its half-maximal inhibitory concentration (IC_50_), SAC was added to the MCR enzyme solution at concentrations of 0, 25, 50, 100, 250, 500, 800, 1,000 and 1,500 µmol/L. The same ELISA procedure described above was followed, and the IC_50_ was calculated using nonlinear regression curve fitting in GraphPad Prism (version 9, GraphPad, La Jolla, CA, USA).

### In vitro fermentation experiment

The experimental procedures involving animals were approved by the Animal Care and Use Committee of the Chinese Academy of Agricultural Sciences (IAS2023-131; Beijing, China). Rumen fluid was collected from three rumen-fistulated Holstein dairy cows (average body weight: 618 ± 100 kg; milk yield: 23 ± 2.8 kg/d; 3 ± 1 of parity) fed a consistent total mixed ration (TMR). The TMR composition (on a dry matter basis) was as follows: corn silage (25.65%), alfalfa hay (18.59%), steam-flaked corn (26.02%), soybean meal (7.43%), cottonseed meal (7.43%), beet meal (5.58%), distillers dried grains with solubles (7.43%), and minerals and vitamins (1.86%). Approximately 2 h after morning feeding, rumen content was collected, placed in prewarmed insulated bottles, and transported to the laboratory within 30 min. The rumen content was filtered through four layers of sterile cheesecloth and mixed with buffer solution at a 1:2 ratio (v/v) under continuous CO_2_ flushing at 39 °C to prepare the inoculum. The buffer solution was prepared according to Liu et al. [30]. An in vitro batch culture system was employed to evaluate the effects of SAC on rumen fermentation. The fermentation substrate was prepared by drying the TMR at 55 °C for 48 h, grinding to pass through a 1-mm sieve, and weighing 0.5 g into each 120 mL fermentation vial. Each vial received 75 mL of inoculum and the corresponding dose of SAC (0, 0.25, 0.5, 1.0, 1.5, or 2.0 mg/g of substrate DM), with SAC added in dissolved form (1 mg/mL, dissolved in water). The system was flushed with CO_2_, sealed with a rubber stopper and aluminum foil, and incubated in a shaking water bath at 39 °C, 60 r/min, for 24 h. Each treatment included 4 replicates and 4 blank vials (inoculum only) for gas calibration. The experiment was repeated three times on separate days.

After 24 h of fermentation, all fermentation vials were immediately removed from the water bath and placed in ice water to terminate the fermentation process. The gas produced during fermentation was collected in airtight aluminum foil bags, and the total gas production was measured by drawing out the gas using a graduated syringe. From each gas bag, a 5 mL sample of gas was taken to measure CH_4_ concentration. The pH value of the fluid sample was measured using a portable pH meter (Seven Go, Mettler Toledo). From each fermentation vial, three aliquots (4 mL each) of the fermentation substrate (solid-liquid mixture) were collected for VFA analysis (stored at −20 °C) and microbial analysis (stored at −80 °C). The remaining fermentation content in each vial was filtered using nylon bags (8 cm × 12 cm, 350 mesh, SL FILTER, Hangzhou, China) and rinsed thoroughly with water until the effluent became clear. The nylon bags were then dried at 55 °C for 48 h to determine dry matter digestibility (DMD). The concentrations of CH_4_ and VFAs were analyzed using a gas chromatograph (8860, Agilent Technologies, CA, USA), following the parameters and procedures described by Liu et al. [30].

### Genomic DNA extraction and sequencing

The microbial DNA was extracted using the HiPure Stool DNA extraction kit (Magen, Guangzhou, China), the extraction process began with mixing the sample with the sample organic lysis buffer, swirling it for 5–10 min for initial cell lysis. Then, the sodium dodecyl sulfate buffer was added and swirled for 15 s, and the mixture was heated at 70 °C for 10 min to enhance lysis. After centrifugation, phosphate saline buffer and absorption solution were added, mixed and allowed to stand for 5 min. The supernatant was collected by centrifugation at 13,000 × *g* for 5 min, mixed with genomic DNA precipitation buffer, and passed through a DNA column for two rounds for centrifugation separation. Subsequently, the DNA column was washed twice with genomic DNA precipitation buffer and genomic DNA wash buffer 2 to ensure the purity of the DNA. The DNA column was centrifuged and dried for 2 min, and DNA was eluted with preheated DNA elution buffer. The extracted DNA was stored at 2–8 °C for short-term use or −20 °C for long-term storage. The extracted DNA samples were tested for integrity by agar gel electrophoresis, and the concentration and purity were tested by NanoDrop microspectrophotometer (NanoDrop 2000, Thermo Fisher Technologies, USA).

The V4–V5 region of the 16S rRNA gene was amplified by primers Arch519F (5′-CAGCMGCCGCGGTAA-3′) and Arch915R (5′-GTGCTCCCCCGCCAATTCCT-3′). The polymerase chain reaction (PCR) amplification procedure followed the method described by Liu et al. [31]. The amplicons were evaluated on 2% agarose gels and purified with AMPure XP Beads (Beckman, CA, USA). The purified amplicons were then pooled and sequenced on the Novaseq 6000 platform using PE250 mode. The raw reads were then deposited in the NCBI Sequence Read Archive (SRA) database, with the accession number SRP516729. Raw reads were filtered with FASTP v0.18.0 to remove sequences containing ≥ 10% ambiguous bases (N), > 50% of bases with quality scores ≤ 20, and adapter-contaminated sequences. Paired reads were merged with FLASH v1.2.11 (≥ 10 bp overlap, ≤ 2% mismatch), and low-quality tags (quality ≤ 3 for ≥ 3 bases) were trimmed, and only tags with ≥ 75% high-quality bases were retained. Operational taxonomic units (OTUs) were clustered at 97% sequence similarity with USEARCH (UPARSE algorithm, v11.0.667), and chimeric sequences were removed with UCHIME. Taxonomic annotation of representative OTU sequences was performed by alignment against the SILVA v138.2, Greengenes2 v2022.10, and UNITE v10.0 databases using the RDP Classifier v2.2.

For metagenomic sequencing, qualified genomic DNA is fragmented into about 350 bp segments by sonication, then undergoes end-repair and A-tailing. Illumina sequencing adapters were ligated using the NEBNext^®^ Ultra™ Kit (NEB, USA) to generate the sequencing library. Next, 300–400 bp fragments were selected, amplified by PCR, and purified to remove impurities. The library's quality, concentration, and size distribution were assessed with an Agilent 2100 Bioanalyzer (Agilent Technologies, CA, USA), and quantified by real-time PCR. Finally, the library was sequenced on an Illumina Novaseq 6000, where both ends of each fragment are read for 150 bp, generating high-quality sequencing data. The raw sequencing reads were deposited into the SRA database, assigned with the unique accession number SRP527714. Raw reads were quality-filtered using fastp (v0.20.0) to remove sequences containing ≥ 10% ambiguous bases, ≥ 50% low-quality bases (Phred ≤ 20), adapter contamination, or read lengths < 50 bp. Clean reads were assembled with MEGAHIT (v1.2.9) using a multi-k-mer strategy (k = 27–127), retaining contigs ≥ 500 bp. Gene prediction was conducted with MetaGeneMark (v3.38), and predicted genes were clustered using CD-HIT (v4.6) at ≥ 95% identity and ≥ 90% coverage to construct a non-redundant gene catalog. Clean reads were aligned to the gene set using Bowtie2 (v2.3.5.1), and gene abundance was calculated with read reassignment via Pathoscope (v2.0.7), removing genes supported by < 2 reads.

Functional annotation was performed using DIAMOND (v0.9.25, e-value < 1e-5) against multiple databases, including Non-Redundant (NR) protein sequence database (2024.07.25), Kyoto Encyclopedia of Genes and Genomes (KEGG, Release 111), evolutionary genealogy of genes: Non-supervised Orthologous Groups (eggNOG, v5.0), Carbohydrate-Active enzymes (CAZy, 2024.07.14), Pathogen Host Interactions database (PHI-base, v4.17), Comprehensive Antibiotic Resistance Database (CARD, v3.3.0), Quorum sensing-related genes, N cycle gene database (NCycDB), S cycle gene database (SCycDB), P cycle gene database (PCycDB), and Methane cycle gene database (McycDB). Mobile genetic elements (MGE) were annotated using BLASTn (v2.6.0) against the MGE database (2017 version; identity and coverage > 40%), and resistance genes were identified using BacMet-Scan (v1.1) with the antibacterial biocide and metal resistance genes database (BacMet, v2.0). Functional abundance data were analyzed and visualized using R packages.

### Statistical analysis

All experimental data were analyzed using the mixed model procedure of SAS (version 9.4, SAS Institute Inc., Cary, NC, USA). The UNIVARIATE procedure was applied to test the normality of the data. The statistical model was as follows:

$$Y_{ijk} = \mu + G_{i} + S_{j} + e_{ij},$$

where *Y*_*ijk*_ is the dependent variable, *μ* is the grand mean, *G*_*i*_ is the treatment group, *S*_*j*_ is the treatment fixed effect (CON vs. SAC), and *e*_*ij*_ is the error term. Multiple comparisons of means were performed using Tukey’s test. Statistical significance was declared at *P* ≤ 0.05, a tendency was considered when 0.05 < *P* ≤ 0.10.

## Results

### Screening of plant extracts

**Fig. 1:**
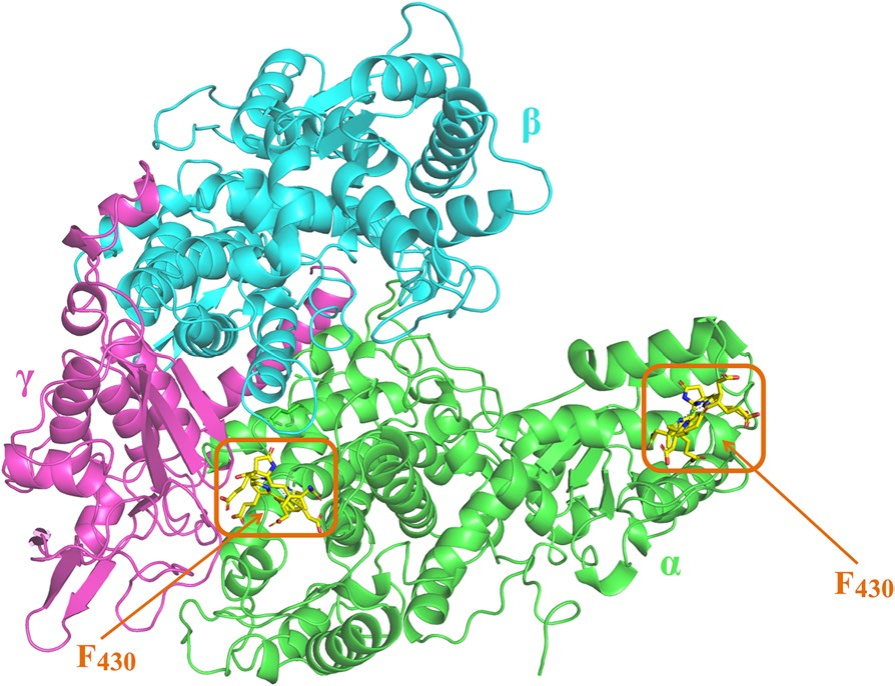
The 3D structure and active sites of MCR. α subunit in green; β subunit in cyan; γ subunit in purple-pink; active site F430 in yellow

**Fig. 2:**
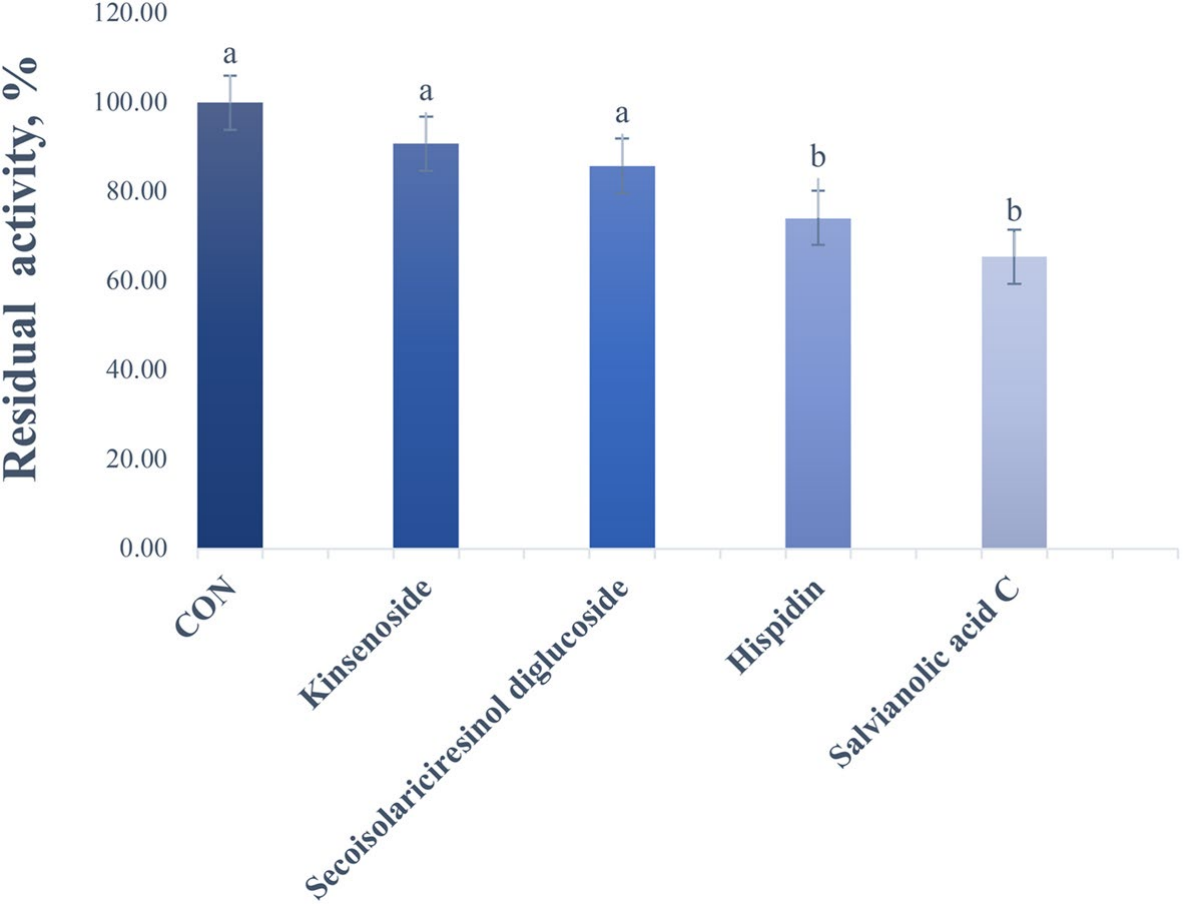
Effects of different plant extracts on inhibition rate of MCR activity. Columns with the same lowercase letters indicate no significant difference (*P* > 0.05), while columns with different lowercase letters denote a significant difference (*P* < 0.05)

The three-dimensional structure of the CH_4_-generating enzyme MCR was modeled, and its active sites (F430 coenzyme-binding pockets) was clearly identified (Fig. 1). Molecular docking was performed to screen plant extracts for potential inhibitors targeting the F430 site. The docking scores, reflecting binding affinity strength, identified four extracts with scores lower than −8 kcal/mol (Table 1). Subsequent in vitro inhibition assays at 100 μmol/L revealed divergent outcomes: Hispidin and SAC significantly inhibited MCR activity by 25.80% and 34.50%, respectively, while the other two extracts showed no inhibitory effects (Fig. 2).

**Table 1:** Molecular docking parameters and details of plant extracts with inhibition of MCR activity

Name	Docking score, kcal/mol	CAS No.	Structure	Mw
Secoisolariciresinol diglucoside	−10.839	257930-74-8		686.70
Kinsenoside	−8.749	151870-74-5		264.23
Salvianolic acid C	−8.124	115841-09-3		492.43
Hispidin	−8.097	555-55-5		246.22

### Molecular binding mode of SAC to MCR

**Fig. 3:**
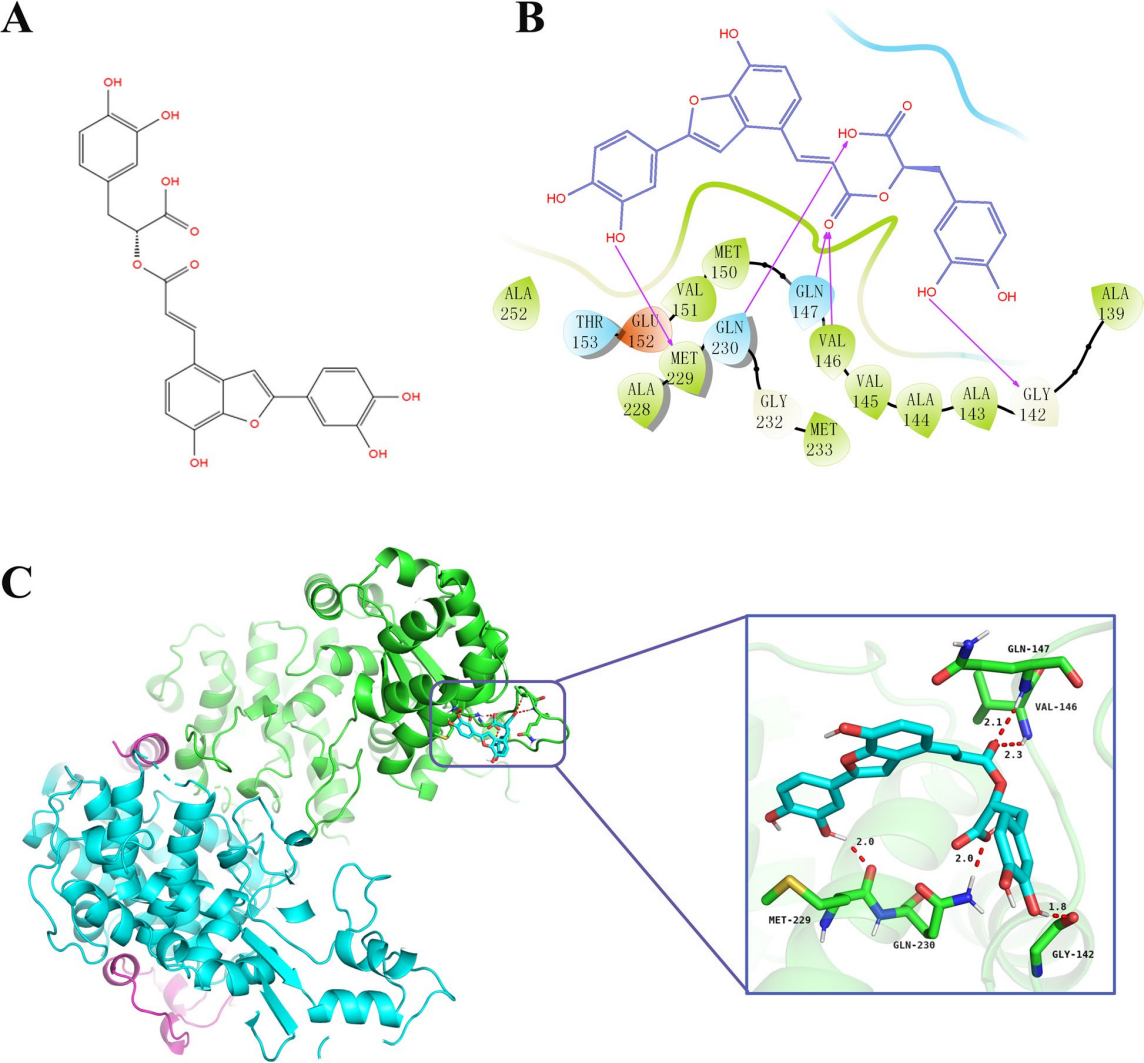
Schematic of SAC and its binding mode with MCR. **A** Chemical structure of SAC. **B** Interaction of SAC with key amino acid residues in the active site of MCR. SAC, blue chemical framework; MCR residues, represented as leaf-shaped markers in different colors; purple arrows indicate hydrogen bond formation. **C** The binding sites and binding modes of SAC and MCR active pockets. The enlarged view shows the binding interaction between SAC and active site residues. The MCR protein C backbone in green; O atoms in bright red, H atoms in white; SAC is represented as light blue sticks; Hydrogen bonds are shown as red dashed lines, with numbers indicating distances (Å)

The chemical structure of SAC is shown in Fig. 3A. The interaction between SAC and MCR was further investigated through molecular docking and high-throughput screening, with a binding free energy of −8.124 kcal/mol (Table 1), indicating a favorable binding affinity between SAC and the MCR active site. This value falls within the range generally considered to represent strong non-covalent binding, as more negative binding free energies correspond to stronger and more spontaneous ligand-receptor interactions. The binding modes of SAC and MCR are illustrated in Fig. 3B–C. In the 3D diagram of McrG, McrB, and McrA subunits, the C-skeleton is displayed in green, the O atoms are shown in bright red, the H atoms are presented in off-white, and SAC is indicated as light blue sticks. The hydrogen bond lengths are represented by red dashed lines. The docking results showed that SAC formed five hydrogen bonds with MCR, with two phenolic hydroxyl groups donating hydrogen bonds to GLY142 and MET229 (1.8 Å and 2.0 Å), a carboxyl group forming a bond with GLN230 (2.0 Å), and an ester group forming two bonds with VAL146 and GLN147 (2.3 Å and 2.1 Å).

### Kinetics of SAC inhibition

**Fig. 4:**
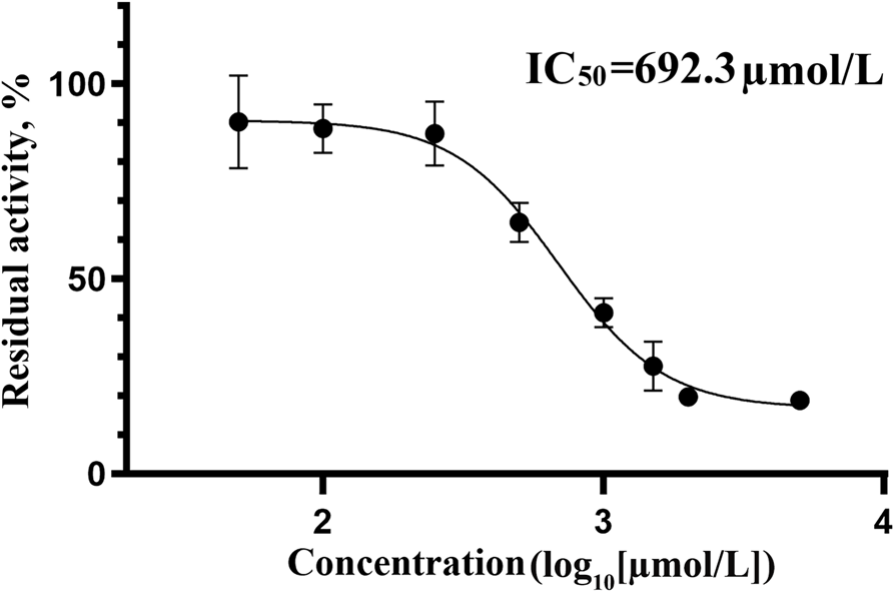
Dose-response curve for the inhibition of MCR activity by SAC. The *y*-axis represents the residual activity of MCR (%), and the *x*-axis represents the log-transformed concentration of SAC (log_10_[μmol/L]). The IC_50_ value, representing the concentration at which 50% of the MCR activity is inhibited. Error bars represent standard deviation (SD) for triplicate experiments

To further evaluate the inhibitory effect of SAC on MCR activity, the activity of MCR was measured under a range of SAC concentrations, and a dose-response curve was plotted (Fig. 4). As shown in the figure, SAC inhibited MCR activity in a concentration-dependent manner. At low concentrations (around log_10_[µmol/L] = 2), the residual activity of MCR remained close to 100%. However, with increasing SAC concentrations, the residual activity of MCR progressively declined. At high concentrations (around log_10_[µmol/L] = 4), the residual activity of MCR was nearly abolished. By fitting the concentration-effect curve, the IC_50_ of SAC on MCR activity was calculated to be 692.3 µmol/L.

### Effects of SAC on CH_4_ production and fermentation in vitro

The effects of SAC on in vitro CH_4_ production, total VFA concentration, and other fermentation parameters in the rumen are shown in Table 2. SAC significantly reduced in vitro CH_4_ production (*P* < 0.01), with reductions of 6.01% to 14.8% compared with the control group. In line with the changes in CH_4_ production, total gas production also significantly decreased with the increasing concentration of SAC (*P* = 0.025). In the 2.0 mg/mL group, the total gas production was 147.5 mL, which was approximately 8% lower than that of the control group (160.3 mL). The addition of SAC did not affect pH value and DMD of rumen fermentation fluid (*P* > 0.05). In terms of VFA concentration, no significant differences were observed between the treatment and control groups (*P* = 0.945). However, in terms of individual VFA components, the proportion of butyrate significantly decreased with the increasing SAC concentrations (*P* = 0.045), whereas acetate and propionate remained unaffected. Additionally, the acetate/propionate ratio showed a decreasing trend across the groups (*P* = 0.096), decreasing from 2.85 in the control group to 2.77.

**Table 2:** Effects of salvianolic acid C on CH_4_ production and other fermentation parameters in vitro

Items	Treatment^1^						SEM	*P*-value
	CON	0.25	0.5	1	1.5	2		
pH	6.97	6.97	6.96	6.95	6.97	6.96	0.007	0.952
DMD^2^	0.79	0.80	0.79	0.79	0.80	0.81	0.212	0.114
Gas production, mg/d	160.3^a^	152.0^b^	149.5^b^	148.8^b^	148.0^b^	147.5^b^	1.250	0.025
CH_4_, mg/d	25.78^a^	24.23^b^	23.55^b^	22.59^b^	21.95^c^	21.97^c^	0.199	0.001
Total VFA concentrations, mmol/L	105.91	106.85	109.12	108.51	107.25	108.68	0.847	0.945
Individual, mol/100 mol								
Acetate	61.78	61.81	61.72	61.81	61.89	61.90	0.515	0.145
Propionate	21.76	21.89	21.47	22.15	22.27	22.35	0.143	0.106
Isobutyrate	1.06	1.05	1.06	1.02	1.02	1.04	0.016	0.174
Butyrate	11.69^ab^	11.55^ab^	11.99^a^	11.41^ab^	11.23^ab^	11.09^b^	0.176	0.045
Isovalerate	2.03	2.01	2.06	1.95	1.92	1.97	0.041	0.379
Valerate	1.69	1.69	1.71	1.67	1.66	1.67	0.023	0.489
Acetate/Propionate	2.85	2.83	2.89	2.79	2.78	2.77	0.013	0.096

^1^*CON *Control (no salvianolic acid C), 0.25, 0.5, 1, 1.5, 2: Salvianolic acid C included at 0.25, 0.5, 1.0, 1.5 and 2.0 mg/g of DM respectively

^2^*DMD* Dry matter digestibility

^a-c^Different superscript letters within the same row indicate significant differences: (*P* < 0.05)

### Impact on rumen microbial communities

**Fig. 5:**
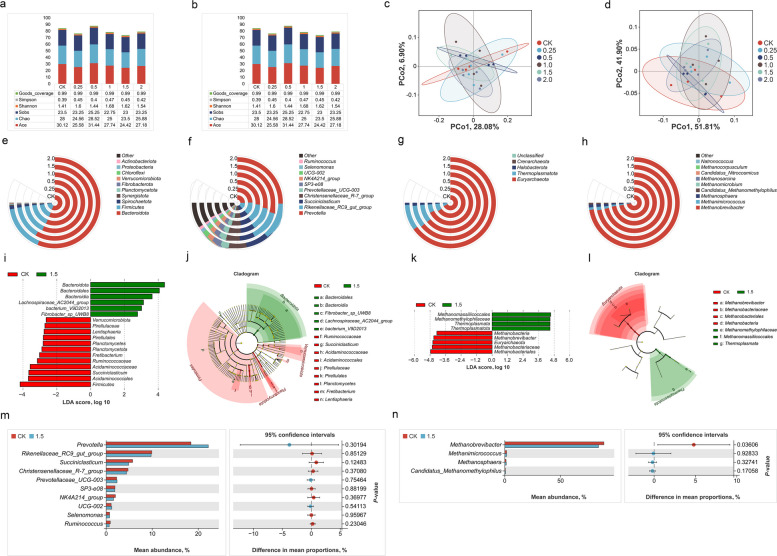
Effects of different concentrations of SAC on rumen microbial community. **a**–**b** Alpha diversity of bacteria and archaea. **c**–**d** Principal coordinate analysis (PCoA) of bacteria and archaea. **e**–**f** Community composition at the phylum and genus levels of bacteria. **g**–**h** Community composition at the phylum and genus levels of archaea. **i**–**j** Linear discriminant analysis effect size (LEfSe) of bacteria. **k**–**l** Linear discriminant analysis effect size (LEfSe) of archaea. **m** Differences in relative abundance of bacteria at genus level (1.5 mg/g DM of SAC). **n** Differences in relative abundance of archaea at genus level (1.5 mg/g DM of SAC)

The alpha diversity indices of bacterial and archaeal communities under SAC treatment were shown in Fig. 5a–b, which evaluated microbial richness and evenness. No significant differences were observed between the SAC treatments and the control group, although slight variations were appeared at certain concentrations. The principal coordinate analysis (PCoA) of bacterial and archaeal communities (Fig. 5c–d) showed the distance between communities. The community structure in the 1.5 mg/g DM SAC treatment group exhibited a distinct shift away from the control. Changes in microbial community composition under different concentrations of SAC were shown for bacteria at the phylum and genus levels (Fig. 5e–f) and for archaea at the phylum and genus levels (Fig. 5g–h). For bacteria, Bacteroidota and Firmicutes were the predominant phyla in the rumen, dominating all treatment groups. At the genus level, *Prevotella*, *Rikenellaceae_RC9_gut_group*, and *Succiniclasticum* were the major bacterial genera. For archaea, Euryarchaeota dominated all treatment groups, and *Methanobrevibacter* was the primary archaeal genus.

The shifts in bacterial and archaeal communities in response to SAC treatment (1.5 mg/g DM) compared with the control group were analyzed using multiple approaches (Fig. 5i–n). The linear discriminant analysis effect size (LEfSe) revealed that bacterial taxa such as Bacteroidota and Bacteroidales were significantly enriched in the SAC treatment group, whereas Firmicutes, Actinomycetales, and Ruminococcaceae were enriched in the control group (Fig. 5i). The corresponding cladogram (Fig. 5j) further illustrated these differences, with green-highlighted branches representing taxa enriched with SAC treatment (e.g., Bacteroidota and Bacteroidales) and red-highlighted branches denoting those enriched in the control (e.g., Firmicutes and Ruminococcaceae). For archaeal taxa, the linear discriminant analysis (LDA) showed that Methanomassiliicoccales and Thermoplasmata were enriched in the treatment group, while Euryarchaeota and *Methanobrevibacter* were more abundant in the control group (Fig. 5k). This was further visualized in Fig. 5l, where green branches highlighted archaeal taxa enriched in the treatment group, such as Methanomassiliicoccales, and red branches showed taxa like *Methanobrevibacter*, enriched in the control group. Statistical analyses of the relative abundances of bacterial and archaeal genera (Fig. 5m and n) supported these findings. The relative abundance of *Prevotella* is numerically greater in the control group, but this difference was not statistically significant (Fig. 5m). In contrast, *Methanobrevibacter*, had a significantly higher relative abundance in the control group compared to the SAC treatment group (*P* < 0.01; Fig. 5n).

### Effects of SAC on functional gene expression and methanogenesis

**Fig. 6:**
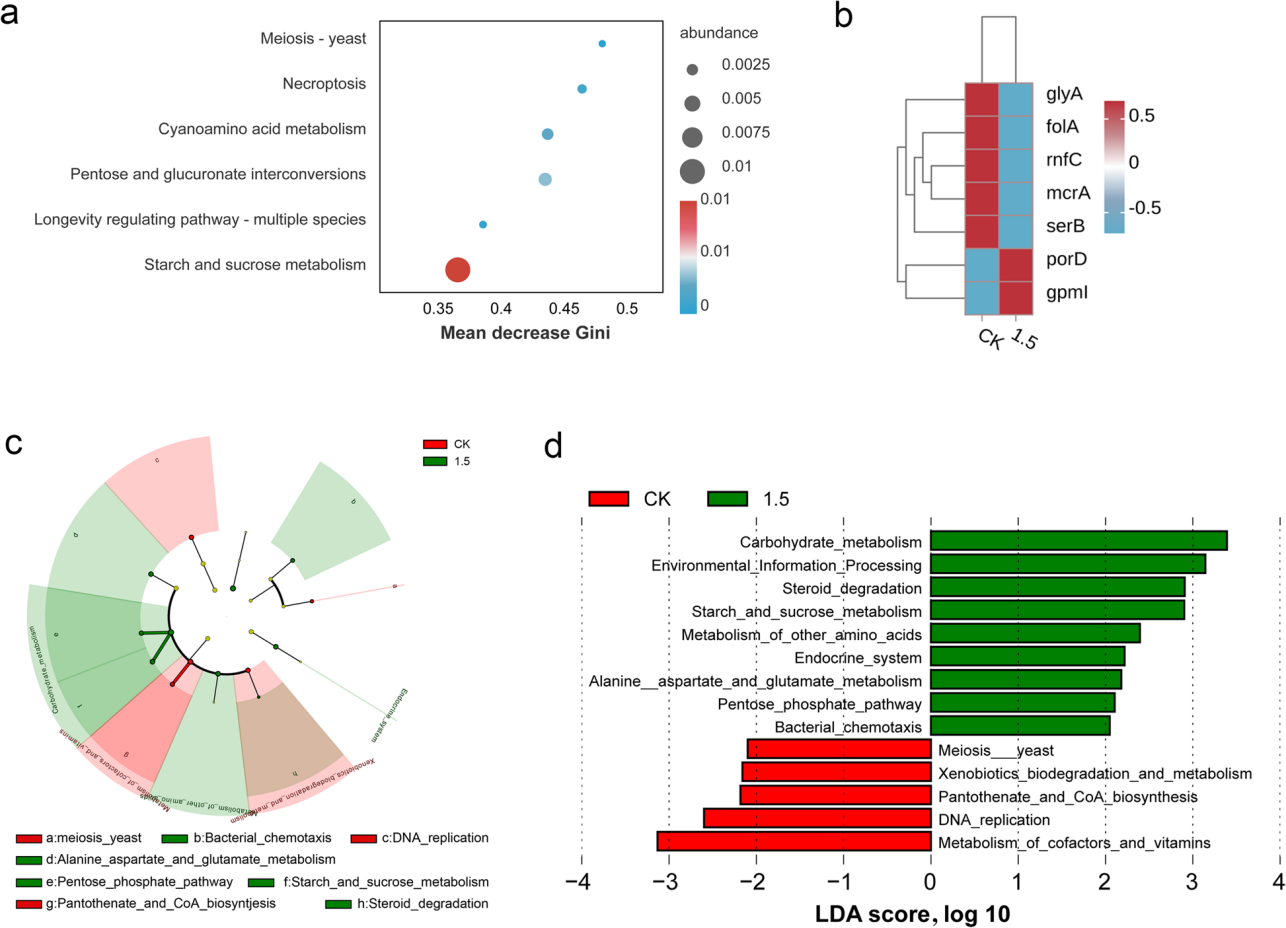
Effects of SAC (1.5 mg/g DM) on metagenomic functional genes of rumen microbiota. **a** Random Forest analysis of functional genes based on KEGG pathway. The horizontal axis represents the Gini index of average impurity reduction, and the larger the Gini index of a species, the more pronounced its ability to distinguish between two groups. **b** Heatmap of methane metabolism related genes. **c**–**d** Linear discriminant analysis effect size (LEfSe) of functional genes based on KEGG pathway

The functional gene profiles with SAC treatment were evaluated using random forest analysis (Fig. 6a). Pathways associated with starch and sucrose metabolism contributed most to the observed differences between groups. The genes involved in steroid degradation were markedly enriched (Fig. 6c–d). The heatmap showed that important methanogenesis genes, such as *mcrA* and *rnfC* (electron transport complex subunit C), were significantly downregulated in the SAC group (Fig. 6b). The LEfSe analysis provided additional evidence of functional shifts (Fig. 6c–d). The cladogram illustrated that starch and sucrose metabolism, steroid degradation and the pentose phosphate pathway were enriched in the SAC treatment group, whereas DNA replication was enriched in the control (Fig. 6c). Consistently, the LDA score plot confirmed that carbohydrate metabolism and steroid degradation were significantly enriched with SAC treatment, while DNA replication was more abundant in the control group (Fig. 6d).

## Discussion

The molecular docking results confirmed that SAC was an effective noncovalent inhibitor of MCR, with a GlideScore of −8.124 kcal/mol. This strong predicted binding affinity is primarily attributed to the formation of five key hydrogen bonds, as illustrated in the 3D interaction model (Fig. 3). The shortest hydrogen bond (1.8 Å) was formed between the phenolic hydroxyl group of SAC and GLY142, a residue situated near the active site of MCR. Although GLY142 has not been explicitly identified as a catalytic residue, its close proximity to the Ni-containing F430 cofactor suggests a possible structural role in ligand stabilization. In addition, hydrogen bonds with lengths shorter than 2.0 Å are generally considered particularly significant, as they tend to exhibit stronger binding interactions than those exceeding 2.5 Å [32]. SAC also formed additional interactions with MET229 (2.0 Å), GLN230 (2.0 Å), and GLN147 (2.1 Å), further stabilizing the compound within the MCR active site. These interactions enhance the binding affinity of SAC, supporting its potential as an effective MCR-targeting CH_4_ inhibitor. Among the plant extracts tested in this study, SAC demonstrated the strongest inhibitory effect on MCR activity, achieving a 34.50% reduction (Fig. 2). Interestingly, although other extracts exhibited comparable predicted binding affinities in molecular docking, they did not produce similar levels of enzyme inhibition. This discrepancy suggests that binding affinity alone may not reliably predict enzyme inhibition, while actual enzyme inhibition is also influenced by other factors such as ligand solubility, conformational dynamics and experimental conditions [33]. Therefore, biochemical assays are essential to validate in silico predictions. To further evaluate the inhibitory efficacy of SAC, the concentration dependent inhibition of MCR by SAC was assessed, and its IC_50_ was determined to be 692.3 µmol/L. This value indicates that SAC is a moderately potent inhibitor of MCR. Although studies directly reporting IC_50_ values are limited, similar to other plant derived polyphenols, relatively high concentrations are generally required to reduce CH_4_ emissions or the expression of MCR genes [27, 34]. Synthetic compounds targeting MCR typically exhibit much lower IC_50_ values, such as 3-NOP (IC_50_ = 0.1 µmol/L) [21] and BES (IC_50_ = 0.4 µmol/L) [24], which are markedly lower than that of SAC. However, the practical application of these synthetic inhibitors has been limited by their requirement for relatively high doses to achieve effective inhibition and potential cytotoxicity concerns [24, 25]. In contrast, plant derived compounds generally offer higher biocompatibility [35]. SAC is a water-soluble polyphenolic compound isolated from the root of *Salvia miltiorrhiza*, a traditional Chinese medicinal herb [36]. It has been reported to possess a wide range of biological activities, including antioxidant, anti-inflammatory, and antimicrobial properties [37]. These properties are not only beneficial in health applications but also suggest the potential of SAC as an environmentally sustainable CH_4_ inhibitor in agricultural systems.

SAC significantly reduced total gas production, and this reduction was more pronounced at higher concentrations, primarily reflecting its inhibitory effect on CH_4_ production. The hydroxyl groups of polyphenols can form hydrogen bonds and hydrophobic interactions with microbial enzymes, thereby influencing binding activity [38, 39]. As a polyphenolic compound, SAC may exhibit similar characteristics, which could help explain its impact on CH_4_ production. Previous studies have shown that plant polyphenolic compounds can selectively reduce CH_4_ emissions by inhibiting methanogen activity, reducing H_2_ availability, or altering rumen microbial community composition [40]. For instance, representative plant polyphenols such as tannins and flavonoids have been reported to reduce CH_4_ production, potentially by inhibiting the activity of methanogens or disrupting the stability of microbial membranes [40–42]. Consistent with these polyphenol mechanisms, our molecular docking analysis indicated that SAC can interact directly with MCR, the key enzyme in methanogenesis, thereby providing molecular evidence for its role in reducing CH_4_ production. Moreover, the antioxidant properties of SAC may further enhance its inhibitory effects on CH_4_ production. Antioxidants have been reported to reduce CH_4_ emissions by modulating rumen fermentation and altering microbial community composition [43].

Although total gas production decreased with SAC treatment, the relatively stable concentrations of VFAs indicated that overall rumen fermentation was not severely disrupted, and the reduction in CH_4_ production may partially reflect selective inhibition of methanogenesis. While H_2_ concentration was not directly measured in this study, previous studies have demonstrated that suppression of methanogenesis can redirect metabolic hydrogen ([H]) toward alternative sinks, such as propionate formation or microbial biomass synthesis, rather than CH_4_ production [27, 44]. In this context, the significant decrease in butyrate proportion (*P* = 0.045) and the trend toward a lower acetate/propionate (A/P) ratio (*P* = 0.096) may suggest shifts in [H] distribution. Because butyrate formation is generally associated with the release of H_2_ and propionate formation consumes it, these observations may reflect a redistribution of [H] away from methanogenesis [45, 46]. Future research should incorporate H_2_ monitoring to validate the proposed mechanism by which SAC alters the destination of [H] flux.

The pH values and DMD were not significantly affected across treatments, and their stability indicates that SAC does not impair the fundamental fermentation processes of the rumen. Maintaining pH within a narrow range (6.95–6.97) is essential for optimal microbial activity, particularly for fiber-degrading bacteria responsible for fiber digestion [47]. In vitro fermentation systems often maintain slightly higher pH values due to the buffering capacity of the medium and limited acid accumulation during short incubation periods, as similarly reported by Ertl et al. [48] in a study of in vitro rumen fermentation. The absence of significant changes in DMD suggests that SAC does not inhibit fiber degradation, consistent with the observation that VFA concentrations remained consistent across treatments. Collectively, these findings indicate that SAC selectively inhibits methanogenesis without adversely affecting other fermentation processes.

SAC also altered the abundance of certain bacterial and archaeal taxa, but overall microbial diversity and community evenness remained unchanged, suggesting that SAC selectively targets specific methanogenic archaea rather than broadly disrupting the rumen microbiota. In the bacterial community, Bacteroidota increased, while Firmicutes and Ruminococcaceae decreased with SAC (1.5 mg/g DM). Bacteroidota are generally considered degraders of non-fiber carbohydrates and potential H_2_ utilizers, whereas Firmicutes, including Ruminococcaceae, are regarded as fiber fermenters and major H_2_ producers [49, 50]. These community shifts suggest that the reroute of fermentation end products may indirectly reduce the substrates available to methanogens.

On the archaeal side, the abundance of *Methanobrevibacter* declined markedly with SAC, while Methanomassiliicoccales increased. *Methanobrevibacter* is the dominant hydrogenotrophic methanogen in the rumen, and its suppression directly reduces CH_4_ production [51, 52]. In contrast, Methanomassiliicoccales are methylotrophic methanogens that use H_2_ as an electron donor to reduce methylated substrates (e.g., methanol) into CH_4_ [53]. The differential response of these two archaeal groups may reflect differences in their substrate utilization pathways and ecological roles rather than inherent differences in MCR structure, which is highly conserved among methanogens [54]. Meanwhile, transcriptome analysis shown that Methanomassiliicoccales are more active in low CH_4_ ruminant hosts [55]. These findings suggest that SAC may contribute to a shift in the archaeal community toward methanogens with lower CH_4_ production efficiency, although further studies are needed to confirm this effect.

Metagenomic analysis revealed that SAC (1.5 mg/g DM) enriched genes involved in carbohydrate metabolism pathways, particularly starch and sucrose metabolism and the pentose phosphate pathway. These pathways can generate additional NADPH and fermentable intermediates, thereby improving energy yields without increasing the availability of H_2_ for methanogenesis [56]. Moreover, SAC enriched the steroid degradation pathway, suggesting that microbes had an increased ability to utilize complex carbon sources. Bacterial steroid degradation is considered an important component of the global carbon cycle, as it converts complex sterol carbon into small molecules that can enter metabolic pathways, thereby promoting carbon recycling [57, 58].

Importantly, SAC downregulated two key methanogenesis genes: *mcrA* and *rnfC*. Downregulation of *mcrA* directly impairs the enzyme that catalyzes CH_4_ formation [59], while downregulation of *rnfC* may limit H_2_ utilization for methanogenesis [60]. In contrast, DNA_replication genes were more enriched in the control group, which may reflect a higher proliferative activity of certain microorganisms, such as *Methanobrevibacter*. Overall, these functional and compositional changes further support the selective inhibition potential of SAC for methanogenesis.

Taken together, evidence from molecular docking, enzyme activity assays, and metagenomic profiling indicates that SAC suppresses methanogenesis primarily by directly inhibiting MCR activity and by downregulating methanogenesis-related genes, particularly in hydrogenotrophic archaea such as *Methanobrevibacter*. However, the mechanism by which SAC enters methanogens to bind MCR remains unresolved. Given its low molecular weight relative to macromolecules, passive diffusion or non-specific transporter mediated uptake are possible routes [61]. Similar uncertainty exist for other MCR inhibitors, as the exact intracellular transport into methanogens of compounds such as BES has not been experimentally confirmed [62]. Further research using targeted uptake and localization approaches will be essential to determine how SAC enters methanogen cells and interacts with MCR.

## Conclusion

This study demonstrates that SAC has potential as a CH_4_ inhibitor in the rumen. SAC significantly reduced CH_4_ production by selectively targeting *Methanobrevibacter*, the dominant methanogen genus involved in hydrogenotrophic methanogenesis. The modulation of the rumen microbiota, particularly the enrichment of beneficial bacterial taxa such as Bacteroidota, suggests that SAC may indirectly inhibit CH_4_ production by altering the H_2_ balance in the rumen. Furthermore, SAC has the ability to reduce the expression of key genes involved in CH_4_ metabolism, including *mcrA* and *rnfC*, highlights its potential to interfere with the enzymatic processes responsible for CH_4_ generation. Importantly, SAC did not adversely affect overall fermentation efficiency, as evidenced by the stable pH, DMD, and VFA concentrations. Collectively, these findings support the feasibility of incorporating SAC as a feed additive to mitigate CH_4_ emissions in dairy cow. Future studies are warranted to optimize its application and to assess its long-term impacts on animal health and productivity.

## Data Availability

The 16S rRNA raw sequencing reads have been deposited in the NCBI Sequence Read Archive (SRA) under the accession number SRP516729. Similarly, the metagenomic raw sequencing reads are available in the NCBI SRA database under the accession number SRP527714. Additional raw data can be obtained from the corresponding author upon reasonable request.

## Abbreviations

3-NOP: 3-Nitrooxypropanol
A/P ratio: Acetate-to-propionate ratio
BacMet: Antibacterial biocide and metal resistance genes database
BES: 2-Bromoethane sulfonate
BPS: 3-Bromopropane sulfonate
CARD: Comprehensive antibiotic resistance database
CAZy: Carbohydrate-active enzymes
CH_4_: Methane
CO_2_: Carbon dioxide
DM: Dry matter
DMD: Dry matter digestibility
ELISA: Enzyme-linked immunosorbent assay
eggNOG: Evolutionary genealogy of genes non-supervised orthologous groups
GHG: Greenhouse gas
IC_50_: Half-maximal inhibitory concentration
KEGG: Kyoto Encyclopedia of Genes and Genomes
LDA: Linear discriminant analysis
LEfSe: Linear discriminant analysis effect size
MCR: Methyl-coenzyme M reductase
*mcrA*: Methyl-coenzyme M reductase subunit α gene
McycDB: Methane cycle gene database
MGE: Mobile genetic element
NADPH: Nicotinamide adenine dinucleotide phosphate
NCycDB: N cycle gene database
NR: Non-redundant
PCoA: Principal coordinate analysis
PCR: Polymerase chain reaction
PCycDB: P cycle gene database
PHI-base: Pathogen host interactions database
*rnfC*: Electron transport complex subunit C
SAC: Salvianolic acid C
SCycDB: S cycle gene database
SI: Supporting information
VFA: Volatile fatty acid
